# Editorial: Mechanisms of stress tolerance in horticultural crops: physiological and molecular insights

**DOI:** 10.3389/fpls.2025.1664603

**Published:** 2025-08-12

**Authors:** Milan Kumar Lal, Ravinder Kumar, Rahul Kumar Tiwari, Awadhesh Kumar, Abazar Ghorbani, Necla Pehlivan, Meisam Zargar

**Affiliations:** ^1^ Division of Crop Physiology and Biochemistry, Indian Council of Agricultural Research (ICAR)-Central Rice Research Institute, Cuttack, Odisha, India; ^2^ Indian Council of Agricultural Research (ICAR)-Central Potato Research Institute, Shimla, India; ^3^ Division of Plant Pathology, Indian Council of Agricultural Research (ICAR)-Indian Agricultural Research Institute, New Delhi, India; ^4^ Division of Crop Protection, Indian Council of Agricultural Research (ICAR)-Indian Institute of Sugarcane Research, Lucknow, India; ^5^ National Key Laboratory for the Development and Utilization of Forest Food Resources, The Southern Modern Forestry Collaborative Innovation Center, State Key Laboratory of Tree Genetics and Breeding, Key Laboratory of State Forestry and Grassland Administration on Subtropical Forest Biodiversity Conservation, College of Life Sciences, Nanjing Forestry University, Nanjing, Jiangsu, China; ^6^ Department of Biology, Recep Tayyip Erdogan University, Rize, Türkiye; ^7^ Department of Agrobiotechnology, Institute of Agriculture, RUDN University, Moscow, Russia

**Keywords:** abiotic stress, horticultural crops, phytohormone, climate change, plant physiology

## Impact of abiotic stress on horticultural crops

The current trend of change in the climate scenario which leads to abiotic stress such as drought, saline, high temperature and heavy metal stress, seriously impacts the cultivation of horticultural crops. The recent report suggests new developments in the area of plant science and biotechnology techniques that can tackle these issues. The Research Topic “Mechanism of Stress Tolerance in Horticultural Crops: Physiological and Molecular Insights”, synthesises recent progress in understanding plant responses, which explores multifaceted approaches to crop improvement. The abiotic stresses, such as drought stress, salinity stress, high temperature (Park et al.) and heavy metal stress substantially reduces the water uptake, which affects the photosynthesis, synthesis of reactive oxygen species (ROS), chlorophyll degradation, and overall plant defence mechanisms (An et al.; Lei et al.; Liang et al.). Plants that face these stresses synthesise reactive oxygen species, like O_2_⁻, H_2_O_2_, and OH⁻, which act as stress marker molecules (Li et al.).

## Decoding the molecular mechanism of plant stress tolerance

### Transcription factors

The prominent area of research focuses on the transcription factors that play an important role in regulating the gene expression in response to various stresses (Zuo et al.). In the recent study, the DELLA protein, which is the key regulator of sweet potato development and stress responses, has been identified as a potential target for improving abiotic stress tolerance. A comprehensive comparative analysis of DELLA genes in six *Ipomoea* species aimed to identify genetic targets for sweet potato improvement (Zuo et al.). A comprehensive evolutionary analysis across six *Ipomoea* species, including sweet potato (*Ipomoea batatas*), identified 20 members of the DELLA protein family. These proteins typically range from 500 to 600 amino acids in length, with molecular weights between 56–65 kDa, exhibiting moderate hydrophilicity (Zuo et al.). Secondly, the TCP gene family members in *Opisthopappus taihangensis* are involved in plant growth and abiotic stress responses. Some genes like *OtTCP4*, *OtTCP9*, and *OtTCP11* are seen as key players for providing tolerance to *O. taihangensis* under abiotic stress (Gao et al.).

Similarly, NAC transcription factors are widely involved in abiotic stress responses, including drought and salinity, and play a significant role in plant growth and development. For example, *HcNAC35* in night lily showed upregulated expression under drought and salinity stress, enhancing stress tolerance in transgenic watermelon. In the same way, *IbNAC3* in sweet potato helps the plant handle salt and drought, and StNAC053 in potato improves salt and drought tolerance in transgenic Arabidopsis (Cao et al.). Similarly, other transcription factors like AP2/ERF, WRKY, GRAS, and NF-YA also emerge as key players in plant stress tolerance. The AP2/ERF transcription factor family includes ERF8 and ERF043, identified in rapeseed’s response to aluminium stress. GRAS transcription factors are also known for their roles in abiotic stress responses. (Li et al.).

### Hormonal signalling

Melatonin is a hormone that is present in plant, which scavenges ROS, RNS and enhances antioxidant activities, which provide tolerance to abiotic stress in horticultural crops (Tiwari et al., 2020, 2021; Altaf et al., 2023). Melatonin has been shown to mitigate heat stress in tomato seedlings by enhancing photosynthetic capacity, protecting PSII, and modulating chlorophyll metabolism (Pehlivan and Guler, 2018). It also alleviates high nitrogen stress in apple rootstocks by maintaining carbon-nitrogen balance and improving root activity, reducing root growth inhibition. In apple rootstock M9T337, melatonin mitigates high nitrogen (N) stress (30 mmol L^−1^), which typically inhibits root growth and disrupts carbon-nitrogen metabolism. In addition, exogenous melatonin (100 mmol L^−1^) significantly improves rootstock growth, including root length, number of root tips, surface area, and volume, along with increased root and leaf biomass (Sun et al.). Similarly, in tomato seedlings, exogenous melatonin application provides protective mechanisms under heat stress, safeguarding chlorophyll metabolism and photosynthesis, reduces oxidative damage indicators such as H_2_O_2_, MDA, and electrolyte leakage, while enhancing the activities of key antioxidant enzymes (CAT, POD, SOD, APX) and improving overall photosynthetic efficiency (An et al.). Other hormones such as auxin, gibberellin (GA), salicylic acid (SA), and ethylene are also involved in complex signalling networks that mediate plant responses to various abiotic and biotic stresses. On the other hand, salicylic acid can protect plants from drought stress by mediating ABA-related gene expression and enhancing antioxidant defence (Pehlivan et al.).

### RNA editing, microRNAs and gene editing

RNA editing, particularly C-to-T conversions in mitochondrial genes like *nad9*, has been identified as a significant mechanism influencing protein structure and enhancing drought resilience in wheat. For instance, failure of RNA editing to improve protein efficiency shows increase in plant susceptibility to abiotic stress (Mohamed et al.). On the other hand, microRNA169 (miR169) is found a highly conserved miRNA that regulates vascular development, water homeostasis, oxidative stress response, and drought tolerance in crops like potatoes. In potato (*Solanum tuberosum* L.), silencing of StmiR169a significantly improves drought tolerance by enhancing vascular architecture, reactive oxygen species scavenging, and photosynthesis. Eight potato pre-miR169 genes (a-h), identified on chromosomes 3, 7, and 8, all produce a single mature miRNA. (Lei et al.). NAD9 RNA editing is a crucial post-transcriptional modification occurring in plant mitochondria, and a study on wheat (*Triticum aestivum*) investigates nad9 RNA editing patterns under drought stress in both tolerant (Giza168) and moderately tolerant (Gemmiza10) cultivars. In the drought-tolerant Giza168, 22 editing sites were identified, with 14 sites significantly altered by drought stress and seven resulting in synonymous amino acid changes (Mohamed et al.).

### Crop-specific stress management strategies

Beyond understanding molecular mechanisms, recent studies suggest developing specific strategies to manage abiotic stress in various crops. Hydrogen-rich water (HRW) has also been explored for its stress-mitigating properties. In strawberry seedlings under salt stress, HRW treatment, prepared by bubbling hydrogen gas into deionised water to saturation and then diluting to specific concentrations, regulated both plant physiological responses and root endophytic bacteria. Furthermore, HRW helped maintain the activities of antioxidant enzymes (SOD, POD, CAT, APX) and influenced the concentrations of essential ion elements (K, Na, Ca, Mg) and phytohormones (IAA, ABA, SA, GA1), all crucial for plant stress responses (Wang et al.). In another experiment, quinoa’s inherent drought tolerance allows for maintaining crop growth and yield even under deficit irrigation (40% ETc). Nitrogen (N) fertilisation also plays a role in yield and quality, with higher N doses (200 kg N ha^-1^) increasing seed saponin content (Pradhan et al.). For grapes (*Vitis vinifera* L.), particularly the Shine Muscat cultivar, secondary salinisation poses a significant challenge, especially in soils with high EC values (>1500 μS cm^-1^) and total soluble solids (>1000 mg kg^−1^). These amendments improve soil physical properties by increasing water-stable aggregates (WR0.25 up to 73.33%) and mean weight diameter (MWD up to 1.75 mm), while also coordinating soil exchangeable calcium (E-Ca^2+^) and magnesium (E-Mg^2+^) ions and stimulating soil enzyme activities (Zhou et al.).

### Technological advancements in crop improvement

Genomic and transcriptomic analyses are fundamental to dissecting complex stress responses. The process involves genome-wide identification, phylogenetic analysis, gene structure analysis, and conserved motif analysis of gene families like DELLA, TCP, NAC, and CLC, using various public databases such as NGDC, Zenodo, Shigen, Ipomoea Genome Hub, TAIR, NCBI SRA, Phytozome13, PlantTFDB, and miRbase (Cao et al.; Ma et al.; Zuo et al.). Bioinformatics tools like BLAST, HMM, IQ-TREE, MAFFT, MEME, GeneDoc, GSDC, Fastp, STAR, Featurecount, TBtools, HISAT2, DESeq2, HTSeq, MapMan, and WGCNA are extensively employed for sequence alignment, variant calling, expression quantification, and network analysis (Mohamed et al.; Pehlivan et al.).

Gene duplication and evolution are also studied using synteny analysis (MCScanX) and Ka/Ks values to explore gene duplication events and infer evolutionary selection pressures. The exact cleavage site of miRNAs on their target mRNAs can be identified using 5′-RNA ligase-mediated rapid amplification of cDNA ends (5′-RLM-RACE). CRISPR/Cas9, an RNA-guided endonuclease, has emerged as a powerful tool for precise gene modifications, enabling the enhancement of stress tolerance and improvement of various crop traits (Gao et al.; Zuo et al.). Moreover, bioinformatics tools for protein structure analysis, such as SWISS-MODEL and PyMOL are employed to predict and visualise the secondary and tertiary structures of proteins. This helps researchers understand how RNA editing or amino acid changes can impact diverse protein function, stability, and ultimately, plant stress resilience (Gao et al.; Ma et al.). On the other hand, quantitative trait locus (QTL) mapping, when combined with WGCNA and RNA-seq, also provides a powerful approach to narrow down candidate genes associated with specific stress-related traits, such as root length, germination vigor, and dry weight under metal stress. This integrated strategy allows for a more targeted identification of crucial genes, avoiding potential loss of relevant information and leading to more effective utilisation of plant germplasm resources (Li et al.).

## Conclusion

The current research topic highlights the complex dynamic nature of plant response to abiotic stresses and the continuous effort which are dedicated to developing tolerant crop varieties. The research topic elucidates the fundamental function of gene families such as DELLA, TC, CLC, miR169, and NAC for leveraging the effect of RNA editing in protein subunits such as NAD9. Moreover, the implementation of the innovative management strategies, which include the application of biochar and cow dung, exogenous melatonin, and hydrogen-rich water, demonstrates the practical approaches to enhance crop productivity and quality under climate change conditions. The integration of advanced genomics, transcriptomics, and targeted genetic engineering approaches, coupled with a growing understanding of plant-microbe interaction and careful consideration of ethical implications which will be instrumental in ensuring agriculture to ensure global food security. The future research must focus on deciphering the integrated signalling network that mediates the cross-tolerance to multiple abiotic stresses. Moreover, there is also an emerging need to explore the epigenetic regulation, such as DNA methylation and histone modification, which might act as a conferring long-term stress memory in horticultural crops. Additionally, a study on plant-microbiome interaction and expanding RNA editing studies across diverse species may unlock novel adaptive mechanisms under a climate change scenario. Field-based validation of molecular findings and development of climate-resilient genotypes through the genome editing tools like CRISPR/Cas9 remains very much crucial to ensure sustainable horticultural productivity.

## References

[B1] Altaf M. A. Sharma N. Singh J. Samota M. K. Sankhyan P. Singh B.. (2023). Mechanistic insights on melatonin-mediated plant growth regulation and hormonal cross-talk process in solanaceous vegetables. Sci. Hortic. 308, 111570. doi: 10.1016/J.SCIENTA.2022.111570

[B2] Pehlivan N. Guler N. S. (2018). Protective effect of a natural ally on simultaneous mild heat and salt episodes in maise seedlings. Acta Physiol. Plant 40, 1–9. doi: 10.1007/S11738-018-2781-X/FIGURES/4

[B3] Tiwari R. K. Lal M. K. Kumar R. Chourasia K. N. Naga K. C. Kumar D.. (2021). Mechanistic insights on melatonin-mediated drought stress mitigation in plants. Physiol. Plant 172, 1212–1226. doi: 10.1111/ppl.13307, PMID: 33305363

[B4] Tiwari R. K. Lal M. K. Naga K. C. Kumar R. Chourasia K. N. S S.. (2020). Emerging roles of melatonin in mitigating abiotic and biotic stresses of horticultural crops. Sci. Hortic. 272, 109592. doi: 10.1016/j.scienta.2020.109592

